# Genomic footprints of a biological invasion: Introduction from Asia and dispersal in Europe of the topmouth gudgeon (*Pseudorasbora parva*)

**DOI:** 10.1111/mec.15313

**Published:** 2019-12-10

**Authors:** Miguel Baltazar‐Soares, Simon Blanchet, Julien Cote, Ali S. Tarkan, Eva Záhorská, Rodolphe E. Gozlan, Christophe Eizaguirre

**Affiliations:** ^1^ GEOMAR Helmholtz Centre for Ocean Research Kiel Germany; ^2^ CNRS Station d'Ecologie Théorique et Expérimentale (SETE) Moulis France; ^3^ UMR5174 (Laboratoire Evolution et Diversité Biologique) CNRS University Toulouse III Paul Sabatier Toulouse France; ^4^ Faculty of Fisheries Muğla Sıtkı Koçman University Kötekli Muğla Turkey; ^5^ Department of Ecology and Vertebrate Zoology Faculty of Biology and Environmental Protection University of Łódź Łódź Poland; ^6^ Faculty of Natural Sciences Department of Ecology Comenius University Bratislava Slovakia; ^7^ ISEM Université de Montpellier CNRS IRD EPHE Montpellier France; ^8^ School of Chemical and Biological Sciences Queen Mary University of London London UK

**Keywords:** admixture, aquatic, biological invasions, population genomics, *Pseudorasbora parva*, selection in the invasive range

## Abstract

Facilitated by the intensification of global trading, the introduction and dispersal of species to areas in which they are historically non‐native is nowadays common. From an evolutionary standpoint, invasions are paradoxical: not only non‐native environments could be different from native ones for which introduced individuals would be ill‐adapted, but also small founding population size should be associated with reduced adaptive potential. As such, biological invasions are considered valuable real‐time evolutionary experiments. Here, we investigated the population structure and adaptive potential of the highly invasive topmouth gudgeon (*Pseudorasbora parva*) across Europe and East Asia. We RAD‐sequenced 301 specimens from sixteen populations and three distinct within‐catchment invaded regions as well as two locations in the native range. With 13,785 single nucleotide polymorphisms, we provide conclusive evidence for a genome‐wide signature of two distinct invasion events, in Slovakia and Turkey, each originating from a specific area in the native range. A third invaded area, in France, appears to be the result of dispersal within the invasive range. Few loci showed signs of selection, the vast majority of which being identified in the Slovakian region. Functional annotation suggests that faster early stage development, resistance to pollution and immunocompetence contribute to the invasion success of the local habitats. By showing that populations in the invasive range have different evolutionary histories, our study reinforces the idea that populations, rather than species, are the units to consider in invasion biology.

## 1 INTRODUCTION

The rate at which species are reported outside their natural distribution boundaries continues to increase (Bock et al., 2015; Simberloff et al., 2013). Because non‐native species can rapidly establish populations in new locations, they can exert a rapid and severe impact on native ecosystems (Ehrenfeld, 2010), yet not necessarily be detrimental to society and economy (Davis et al., 2011). To understand the whole impact of a biological invasion is complex, and evolutionary approaches should aim to characterize invasion pathways as well as the determinants underlying the successful establishment of non‐native species (Colautti, Grigorovich, & MacIsaac, 2006; Cristescu, 2015). It is well known that ecological or demographic factors such as propagule pressure (the number of individual arriving at a non‐native range) or plasticity in key life history traits (e.g., age at first maturity, reproductive success, faster growth) are important to colonization success and establishment of populations (Britton & Gozlan, 2013; Simberloff, 2009). The role of evolutionary mechanisms such as drift, selection, or adaptive phenotypic plasticity, on the other hand is less well understood (Bock et al., 2015). However, well documented cases do exist, including that of the invasive cane toad (*Bufo marinus*) in Australia, where the rapid evolution of adaptive plasticity facilitated dispersal success (Rollins, Richardson, & Shine, 2015). The use of genetic tools is often applied to infer colonization processes and identify source/sink relationships between native and non‐native range (Estoup & Guillemaud, 2010). The use of genomics in the context of biological invasion is still in its infancy, thus leaving unanswered questions about the role of fine‐scale population structure, adaptive potential or hybridization as a predictor of invasion success. In this work, we intend to fill in this knowledge gap by investigating the genome‐wide signature of one of the most successful cases of biological invasions worldwide, that of the topmouth gudgeon (*Pseudorasbora parva*).

This small freshwater cyprinid was introduced as a byproduct in several countries surrounding the Black Sea in the early 1960s (Gozlan et al., 2010). Chinese carp imports for aquaculture purposes acted as a transport vector from China to Europe. Romania, Hungary, Lithuania and Ukraine are amongst the first sites where it was introduced (Gozlan, 2011; Gozlan, Pinder, & Shelley, 2002). The subsequent establishment of invasive topmouth gudgeon populations occurred at an extremely rapid rate: westward dispersal promoted by human activities, such as translocations of farm fish, recreational fishing or ornamental fish trade, introduced fishes from Hungary into former Czechoslovakia and Germany (1970s); southwards dispersal into Turkey occurred from an introduction point in the Black Sea most likely Bulgaria (Gozlan et al., 2010). First detections in western Europe date back as late as the 1970s, but colonization was only reported in the 1990s (Gozlan et al., 2010). As a result, in 50 years, the distribution of topmouth gudgeon in its invasive range spanned Central and Western Europe, the British Isles and the Turkish territory in Asia Minor and Central Asia. The successful spread of topmouth gudgeon populations in the new Eurasian range is hypothesized to be facilitated by the high plasticity of life history traits, such as short generation time, multiple spawning or small body sizes and reproductive biology that ensures a high reproductive success from as early as the first year of life (Britton, Davies, & Brazier, 2008; Rosecchi, Thomas, & Crivelli, 2001; Yan & Chen, 2009). The species also presents highly plastic responses to a series of environmental factors, such as temperature, population density, physical alterations of the habitat and predator density (Gozlan et al., 2010). The successful establishment of invasive populations of topmouth gudgeon had a series of negative impacts on native ecosystems. For example, the high densities exert intense competition for food resources with native fauna that shares the same trophic niche (Britton, Davies, & Harrod, 2010).

Topmouth gudgeon are also healthy carriers of the intracellular pathogen Rosette agent *Sphaerothecum destruens*, a deadly protist, which is virulent to a large number of European native freshwater fish species (Andreou & Gozlan, 2016; Combe & Gozlan, 2018).

Molecular studies on topmouth gudgeon populations have to date focused on the characterization of the introduction and dispersal pathways in Eurasia. Attempts to understand the invasion pathways from genetic signatures have used mitochondrial DNA (Simon et al., 2011), microsatellites (Simon, Gozlan, Britton, Van Oosterhout, & Hänfling, 2015) or both (Hardouin et al., 2018). The native topmouth gudgeon populations are composed of four deep mitochondrial lineages, two of which constitute the mitochondrial genetic background of all non‐native populations in Europe (Simon et al., 2011). Those lineages originating from China represent a phylogeographic break promoted by the Qinling mountains (Hardouin et al., 2018). At the nuclear level, populations in the native range are more genetically diverse, an observation explained by traditional activities of translocation performed in China over the last 2,000 years (Hardouin et al., 2018). The complex population structure and high diversity observed in the native range of the species suggests, from a molecular perspective, an equally complex invasion scenario offering a unique opportunity to investigate an invasive species’ adaptive potential. Increasing sequencing depth enables covering larger portions of the genomes and clarifying background genomic differentiation while at the same time increasing the chance of identifying candidate loci under selection. In this study, we performed restriction‐associated DNA tags sequencing (RADseq) on specimens collected from 16 populations in Slovakia, Turkey and France representing a chronological gradient (from known records) of invasion. We also added two populations from the native range, identified by Hardouin et al. (2018) as representatives of the two putative invasive mitochondrial lineages.

By screening the genome‐wide diversity of topmouth gudgeon along invaded regions, we (a) characterized the invasion process and hypothetical dispersal in the invasive range; (b) identified candidate loci under selection and (c) characterized their distribution to determine their role in the invasion success of this species. We hypothesize that successful establishment was facilitated by the same loci evolving in parallel across non‐native regions.

## 2 MATERIALS AND METHODS

### 2.1 Sampling scheme of invaded regions

**Figure 1:**
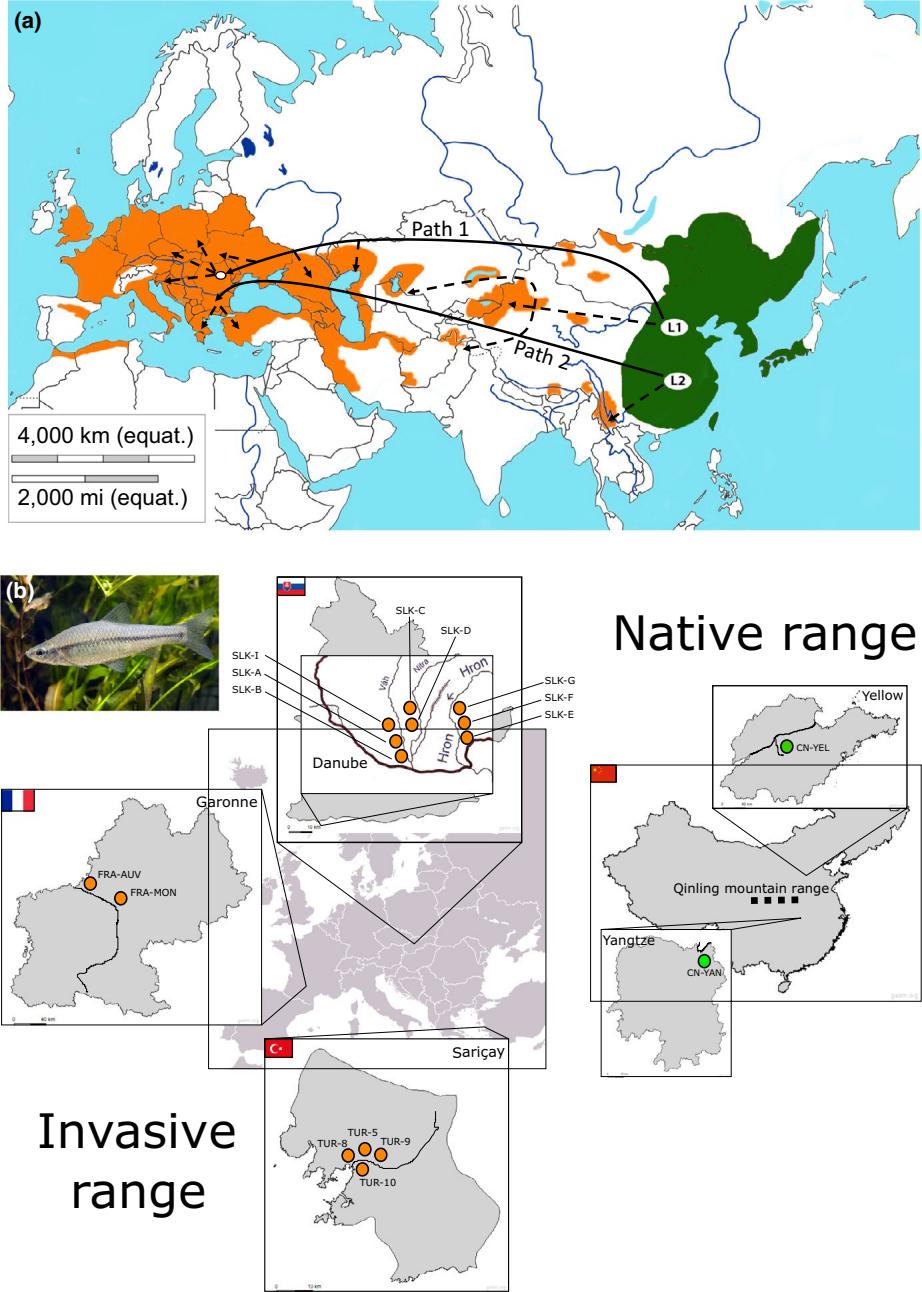
Geographic distribution, introduction pathways and sampling sites. (a) Geographic distribution of *Pseudorasbora parva* in native and invasive range edited from the original Figure 2 of Combe and Gozlan (2018). Identified mitochondrial lineages are represented by L1 and L2. Solid lines indicated the major introduction pathways and dashed the other introductions. Picture used with permission from Wiley. (b) Locations sampled for this work are indicated with green dots in native (China) and orange dots in invasive range (Slovakia, Turkey and France). Major rivers part of the catchment targeted for sampling are depicted in bold lines within each region, as well as the Qinling mountain range [Colour figure can be viewed at http://wileyonlinelibrary.com]

A total of 301 topmouth gudgeon were collected for this study. Samples were distributed across four geographic areas, one in the native range and three in the invasive range, resulting in a total of 16 locations, hereafter populations. In the native range, we used fish collected from the two major catchments in China: the Yangtze (CN‐Yan) and Yellow river (CN‐Yel). Fish from these locations had their mitochondrial DNA partially sequenced and showed highly distinct lineages (Hardouin et al., 2018). In the invasive range, we collected fish from eight sites within the Danube catchment ‐ from two tributaries in Slovakia: three sites from the Vah main catchment, two sites from a branch of the Vah that passes through Nitra and three other sites from the Hron; from four sites in the Sarçay stream, Muğla, Turkey and from two sites from the Garonne catchment in France (Table 1). This way, we aimed to analyse three distinct regions of the invasion range (Figure 1).

**Table 1:** Population information and diversity indices

Population	Range	Region	Latitude	Longitude	*n*	*Ho*	*pA*	*vS*
SLK‐A	Invasive	Slovakia	48.1869	17.7123	17	0.0008	–0.0913	2.7981
SLK‐B	Invasive	Slovakia	48.1326	17.7652	20	0.0009	–0.0663	2.7689
SLK‐C	Invasive	Slovakia	48.1674	18.0627	20	0.0009	0.0757	2.7812
SLK‐D	Invasive	Slovakia	48.1359	18.0197	20	0.0008	0.2958	2.7944
SLK‐E	Invasive	Slovakia	47.9364	18.6304	18	0.0008	–0.2130	2.7122
SLK‐F	Invasive	Slovakia	47.9695	18.5687	18	0.0009	–0.1231	2.7516
SLK‐G	Invasive	Slovakia	48.0270	18.4670	21	0.0009	0.0855	2.7170
SLK‐I	Invasive	Slovakia	48.2695	17.6526	17	0.0008	–0.0561	2.7775
TUR−5	Invasive	Turkey	37.3438	27.7290	15	0.0008	–0.9954	2.3614
TUR−8	Invasive	Turkey	37.3286	27.7127	34	0.0008	–0.9028	2.3312
TUR−9	Invasive	Turkey	37.3509	27.7494	36	0.0008	–0.8518	2.5062
TUR−13	Invasive	Turkey	37.3105	27.7119	7	0.0008	–1.3167	2.3390
FRA‐MON	Invasive	France	43.9462	1.1715	13	0.0007	–0.0902	2.1468
FRA‐AUV	Invasive	France	44.0741	0.8991	30	0.0009	0.5032	2.3011
CN‐YAN	Native	China	29.1500	113.1100	5	0.0006	0.7165	1.7826
CN‐YEL	Native	China	34.8100	117.1200	10	0.0007	1.2237	2.2103

Note

Sampled sites are coded for the tags used in the manuscript.

Abbreviations: *n*, number of samples; *Ho*, observed heterozygosity; *pA*, private alleles; *vS*, variant sites.

### 2.2 DNA extraction, library preparation and restriction‐associated DNA tag sequencing

All fish samples were stored in 90% ethanol prior to extraction. Genomic DNA was extracted with Qiagen DNeasy Kit Blood and Tissue kit (Hilden, Germany) following the manufacturer's instructions. Library preparation, sequencing and bioinformatic processing of raw reads (demultiplexing of individual barcodes, removal of adaptors and barcodes and trimming) was performed at the GeT‐PlaGe core facility, INRA Toulouse, France. Libraries preparation followed Baird et al. (2008). Libraries were constructed by pooling 48 samples after individual barcoding. Samples were digested with *SbfI* restriction enzyme and individually barcoded. The sample pools were sonicated, size selection was performed using SPB beads with adaptors being ligated prior to sequencing. Ten PCR cycles were used to amplify libraries. Library quality was assessed using an Advanced Analytical Fragment Analyser and libraries were quantified by Quantitative PCR using the Kapa Library Quantification Kit. Sequencing took place on an Illumina HiSeq3000 using a single‐end read length of 150 bp Only reads with *Q* > 30 were used in this study.

### 2.3 Filtering and processing of RADseq data

Since there is no reference genome of the topmouth gudgeon, we conducted a de novo assembly of the short reads. To inspect data and chose a robust set of SNPs for downstream analyses, we processed clean reads in several combinations of key de novo assembly parameters with stacks v1.48 (Catchen, Hohenlohe, Bassham, Amores, & Cresko, 2013). We used stacks flexible workflow to investigate how different combination of parameters (minimum depth of coverage, *m*; maximum number of nucleotide mismatches, *M*; number of mismatches between catalogues, *n*) would influence the number of catalogue loci. The objective was to achieve a balance between under merging stacks due to low *m* and *M* and over merging stacks due to high *m* and *M* (Catchen et al., 2013). Ideally, we expected not only the number of loci in the catalogue but also the average stack coverage to flatten at a certain combination of *m*, *M* and *n*. To this end, we first randomly chose five individuals per location and grouped them together as a subsampled data set. We ran all possible combinations of *m* = 2, *m* = 4 and *m* = 6 and *M* = 2, *M* = 5, *M* = 8 while keeping *n* = *M*, as suggested by (Rochette & Catchen, 2017). Variation in average stack coverage and number of loci in the catalogue was inspected graphically for all combinations with r plots made in rstudio. All catalogues of loci were rebuilt after correction with rxstacks.

The complete data set was then processed with the chosen parameter's combination of *m* = 3, *M* = 4 and *n* = 4. To avoid overrepresentation of rare loci, the catalogue of loci formed with the subsampled data set was used as reference (Rodríguez‐Ezpeleta et al., 2016). At the populations step, we followed the 80% rule suggested by Rochette & Catchen (2017) for the parameter *r*. Only one SNP per loci was kept avoiding linkage disequilibrium bias in downstream analyses.

To identify candidate loci under selection in each of the three geographic regions of the invasive range, we created a subset of SNPs per region, hereafter *rSNPs*. This was performed by running stacks pipeline with fish from Slovakia, Turkey and France separately, but matching SNPs against the corrected catalogue loci. While we acknowledge we may have overlooked region‐specific loci, grouping populations per region prior to outlier detection scans is in line with our hypothesis that successful establishment was facilitated by the same loci evolving in parallel across non‐native regions. Importantly, we intended to relax the violations to baseline assumptions common to currently outlier detection methods: low overall differentiation, symmetric migration rates and similar evolutionary and demographic history (Lotterhos & Whitlock, 2014). Lastly, all data sets were filtered out for loci with more than 20% missing data with vcftools (Danecek et al., 2011).

### 2.4 Genetic diversity estimates – comparisons between invasive and native ranges

We calculated the observed heterozygosity (*H_o_*), number of private alleles (*pA*) and number of variant sites (*vS*) in stacks. To account for possible confounding effects linked to individual coverages (Trucchi et al., 2016), genetic diversity indices were tested in a linear model that included the individual coverage and range as explanatory variables: *fit = aov*(*diversity estimate ~ coverage + range*). Models were corrected for the use of sequential sum of squares analyses using *drop1(fit, ~., test = "Chisq")*. Absolute values of *pA* and *vS* were standardized for the depth of coverage at each population, and log transformed to normalize the distribution prior to statistical analyses.

Afterwards, we performed ANOVAs to compare diversity estimates between ranges and among regions. Here we define “range” as native and invasive and “region” as Slovakia, Turkey, France and China, under the null hypothesis that the native range harbours a higher genetic diversity. Note that to investigate the role of range and region in this work, two independent ANOVAs had to be performed. This is because the native range includes one region only. All statistical analyses were performed in r 3.4.1 (R Core Team, 2017).

### 2.5 Population structure at the invasion front and relationship with native sites

To reconstruct the pathway of the topmouth gudgeon's introduction, investigate invasive range expansion and identify candidate loci associated with invasion success, we determined the genetic structure across the species’ spatial distribution. Population structure was investigated by a multifold approach. We started by visually inspecting the distribution of pairwise *F*_ST_, obtained with stacks, in a heatmap and with a hierarchical cluster analysis on the between‐individual genetic distance dissimilarity matrix – visualized in a dendrogram – using the Bioconductor's package snprelate (Zheng et al., 2012). We also investigated the distribution of molecular variance at two maximum hierarchic levels, with AMOVAs (10,000 permutations) implemented in arlequin version 3.5 (Excoffier & Lischer, 2010). The objective was to identify the higher *F*_CT_ between the proposed groupings, as it would indicate the most likely partitioning of molecular variance. The groups tested were: Group I Slovakia‐Turkey‐France‐China (Yellow River)‐China (Yangtze river); Group II Slovakia‐Turkey‐France‐China (Yellow River) and China (Yangtze river); Group III European‐Asian; Group IV Slovakia and Turkey‐ France‐China (Yellow River) and China (Yangtze); Group V Slovakia and China (Yellow River) – Turkey and China (Yangtze river) – France; Group VI Slovakia and China (Yangtze River) – Turkey and China (Yellow river) – France. Pairwise population differentiations were also analysed in arlequin (10,000 permutations).

We then used faststructure (Raj, Stephens, & Pritchard, 2014) to infer the likelihood of fine‐scale population structure. Three independent iterations for values of *K* ranging from 1 to 17 were performed. The most likely number of *K*s was assessed with the algorithm chooseK (Raj et al., 2014). Visualization of admixture proportions was done by constructing membership probabilities plots with the r package adegenet (Jombart, 2008). In order to obtain information on allele frequency divergence between native/non‐native regions, we used structure v2.3.4 (Pritchard, Stephens, & Donnelly, 2000) with *K* = 2 for the following pairwise comparisons: Yangtze River/France, Yangtze River/Turkey, Yellow River/France and Yellow River/Slovakia. Conditions were set to default with a MCMC length of 5 × 10^6^ generations and burnin of 5 × 10^5^. Three independent iterations were performed. We used an admixture model where *a*, the degree of admixture, was inferred from a uniform prior, with initial *a* = 1, max = 10.0 and *SD* = 0.025. The frequency model was set to correlated allele frequencies amongst populations. Comparison of average allele frequency divergences was performed in r.

We then performed a discriminant analysis of principal components (DAPC) to clarify sublevels of population structure. While DAPC is a multivariate method that maximizes genetic differentiation between predefined groups, we rather explored the versatility of *find.clusters* function by removing a priori population assumptions, but restraining the search for *K* to a maximum of 16 (total number of our populations). Best‐fit number of clusters were verified by Bayesian Information criteria (BIC). DAPC is implemented in the r package adegenet (Jombart, 2008). Upon identification of the best‐fit model, we investigated which location, within each region, was probably the most recently colonized. For that, we compared pairwise *F*_ST_ between sampled locations and putative source, under the assumption that higher *F*_ST_ would be a proxy for older introduction time.

### 2.6 Detection of hybrid classes among and within invasive lineages

With the original data set, we investigated the possibility of hybrids (F1, F2 and backcrosses) between lineages as present in the native range. The objective was to test whether hybridization can contribute to the successful colonization in the invasive range. This was done with the software newhybrids v1.1 (Anderson & Thompson, 2002).

### 2.7 Scanning for candidate loci under selection

Outlier detection methods were independently applied to all combined populations within each region. We used the outflank v0.2 which separates the variance caused by (a) the existence of a finite real number of demes, (b) sampling a finite number of individuals per deme and (c) spatial selection from a spatially heterogeneous selection on a specific locus (Whitlock & Lotterhos, 2015). Due to the recent evolutionary timeframe of the invasion, we expect that loci hypothetically under selection occur at lower frequencies. Therefore, we relaxed the filtering step to 40% of missing data per locus and considered a *Hmin* of 0.05.

### 2.8 Environmental correlations

Water velocity in rivers is a factor that might favour the establishment of fish populations, and particularly those of the topmouth gudgeon a species that prefer lentic conditions for reproduction (Boltachev, Danilyuk, Pakhorukov, & Bondarev, 2006). Because the Slovakian sampling sites were distributed between two separate tributaries, the river Váh, with an average river discharge of 196 m^3^/s and the river Hron, with an average discharge of 57.3 m^3^/s, it was possible to investigate if current speed acts as a selective pressure to the establishment of topmouth gudgeon populations. The rationale is that loci highly ranked in Bayes Factor (BF) are possibly affected by the presence of outliers. Hence, candidate loci whose allelic frequencies covary with environmental factor would be suggestive of selection. Environmental correlations were investigated on Slovakia's regional SNP panel and performed in bayenv2 (Günther & Coop, 2013). BF threshold for positive correlations was defined by BF > 10 (Jeffreys, 1998).

### 2.9 Outlier distribution across invasive fronts and relationship with the native range

All statistics were computed in r 3.4.1 (R Core Team, 2017). We compared the list of candidate loci detected in each region to investigate the parallel occurrence of candidate loci under selection. Furthermore, we compared the observed heterozygosity between neutral and candidate loci and further explored how range (native vs. non‐native) and region (China, Slovakia, France, Turkey) affected estimate variation. For that, we built two linear models: in the first, we used “range” and “loci type” (neutral, candidate) as predictors. On the second, we used “region” and “loci type” as predictors. We then performed an ANOVA to compare models and select the best fit model. Lastly, because candidate loci had been used as discriminants of fine‐scale population differentiation (Teske et al., 2019), we investigated signatures of population structure using only candidate loci under selection – applying the same methodology with *F*_ST_ estimates, DAPC analyses and structure.

### 2.10 blast and gene ontology terms of candidate loci

Candidate loci were blasted against ensembl database of annotated genomes, and if no results, again blasted against ncbi database. The search was restrained to the available genomes of fishes. We filtered out low complexity regions and defined sensitivity for short sequences. Meaningful hits were defined as those that overlapped at least 75 bp against the database while reporting an *E*‐value < 10^–4^ (Altschul, Gish, Miller, Myers, & Lipman, 1990). Gene ontology terms (GO) of each successful hit from ENSEMBL were recorded.

## 3 RESULTS

### 3.1 Sequencing statistics and variant calling

A total of 301 fishes were used in this study. On average, 1.3 × 10^6^ reads (*SE* ± 1.31 × 10^5^ reads) were used per individual. After inspection of coverage and number of loci, we kept the combination *m* = 4, *M* = 5 and maintaining *M* = *N* as parameters (Figures S1 and S2). The average individual coverage was 28.7× (*SE* ± 2×). Coverage metrics divided by region and range are presented in Figures S3 and S4. The total number of loci kept after all filtering steps was 13,768.

### 3.2 Genetic diversity estimates in native and invasive range

**Figure 2:**
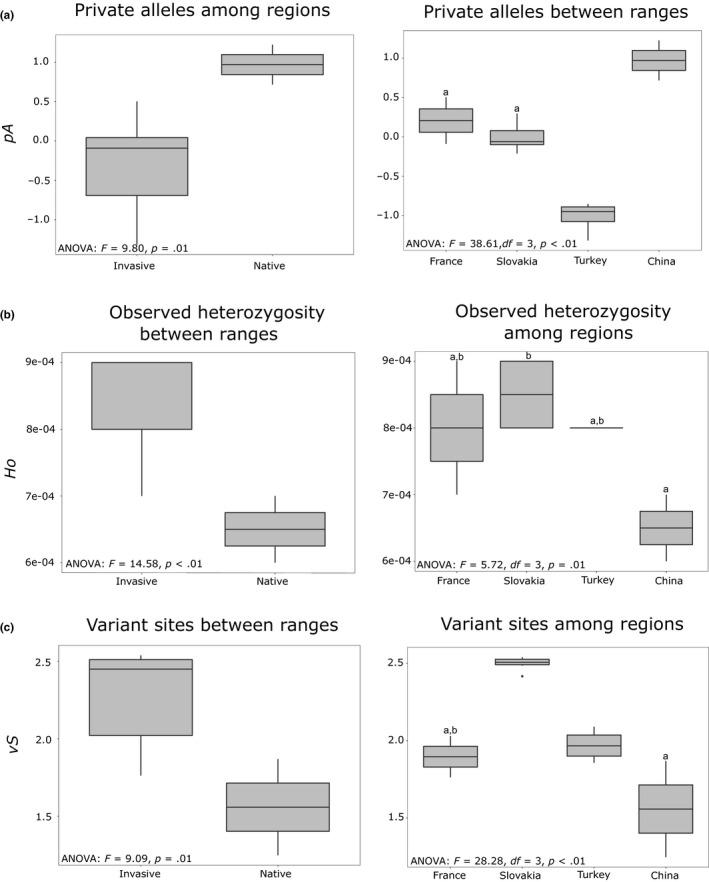
Diversity indices between ranges and among populations (a, b and c). (a) Comparisons of the variance in number of private alleles, *y*‐axis (*pA*), between ranges and among regions; (b) Comparisons of the variance in observed heterozygosity, *y*‐axis (*Ho*) between ranges and among regions; (c) Comparisons of the variance in number of detected variant sites, *y*‐axis (*vS*) between ranges and among regions. Shared letters on boxplots represent nonstatistical significance after performing pairwise multiple comparison with Tukey's HSD

Comparisons of diversity estimates revealed that populations in the invasive range had on average, a lower number of private alleles (ANOVA: *F* = 9.80; *df*_1,14_; *p* = .01) but a higher observed heterozygosity (ANOVA: *F* = 14.58; *df*_1,14_; *p* < .01) and number of variant sites (ANOVA: *F* = 9.09; *df*_1,14_; *p* = .01) than those in the native range (Figure 2). Across regions, i.e., Slovakia, Turkey, France and China, private number of alleles, observed heterozygosity and variant sites varied significantly (ANOVA_pA_: *F* = 38.61; *df*_3,12_; *p* < .01; ANOVA*_Ho_*: *F* = 5.72; *df*_3,12_; *p* = .01; ANOVA*_vS_*: *F* = 28.28; *df*_3,12_; *p* < .01): More specifically, Chinese populations showed on average, higher number of private alleles (Tukey HSD: China vs. France, *p* = .02; China vs. Slovakia, *p* < .001; China vs. Turkey, *p* < .001). Regarding observed heterozygosity, Slovakian populations harboured, on average, higher observed heterozygosity than those in China (Tukey HSD: *p* = .006). Lastly, Slovakian populations showed the highest number of polymorphic sites (Tukey HSD: *p* = .001, Figure 2). None of these metrics correlated with the coverage obtained in each population (Table S1).

### 3.3 Population structure, pathways of invasion and hybridization

**Figure 3:**
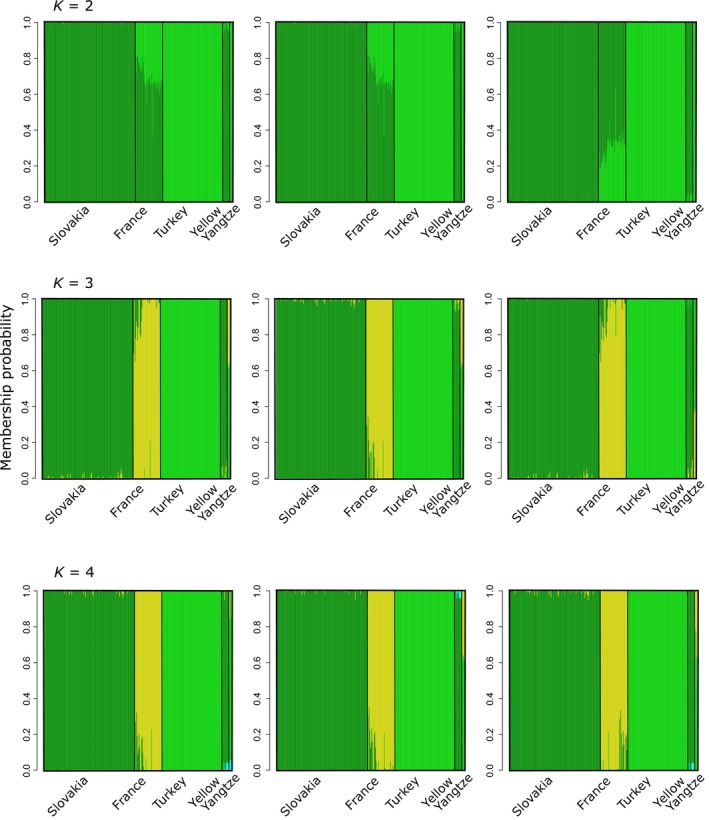
Admixture proportions of native and invasive sampled regions plot. Admixture was estimated for model complexities of *K* = 2, *K* = 3 and *K* = 4, here plotted the three replicates performed with all data set for each value of *K*. In the *x*‐axis is shown the regions from which individuals were taken while the *y*‐axis illustrates the admixture proportion [Colour figure can be viewed at http://wileyonlinelibrary.com]

The population structure of sampled sites investigated with faststructure revealed the likely *K* to range between three and four, but visual representation of membership probabilities suggested *K* = 3 to be a more robust outcome (Figure 3). For *K* = 3, French populations appeared as a distinct cluster, with Slovakia and Turkey belonging to the same cluster of the Yellow River and the Yangtze, respectively.

**Figure 4:**
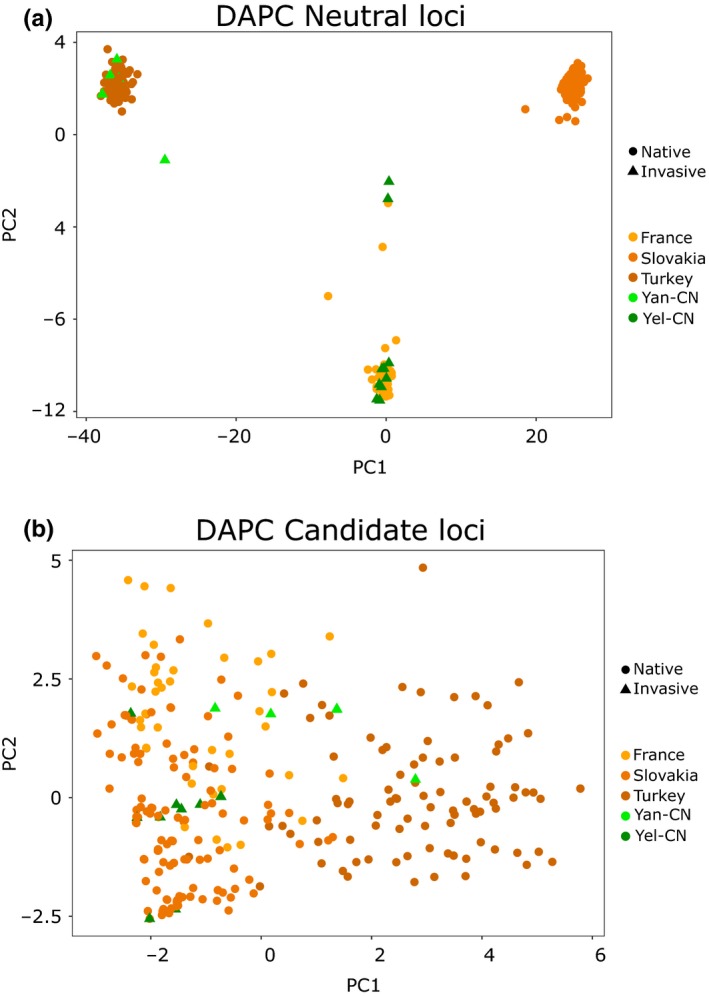
Discriminant analyses of principal component**.** Visual display of the DAPC performed for neutral and candidate loci in order to identify genetic clusters. (a) DAPC was performed for neutral loci and (b) with candidate loci only [Colour figure can be viewed at http://wileyonlinelibrary.com]

Allelic divergence between tested range pairs revealed that Slovakian populations and that of its putative source, the Yellow River, were more genetically similar than any other native/non‐native pairs and that French populations were more similar to those of the Yellow River rather than those of the Yangtze (Figure S6). Discriminant analysis revealed three clusters (retaining 200 PCs and plotting two loadings Figure S7a), formed by Turkey and Yangtze River (China), France and Yellow River (China) and Slovakia (Figure 4a). The analysis of molecular variance (AMOVAs) supported a three‐group partition but with the Slovakian populations grouped with the Chinese population of the Yellow river versus. the Turkish populations grouped with the Chinese population of the Yangtze river and the French populations on their own (*F*_CT_ = 0.296, *p* < .001).

Pairwise *F*_ST_ showed that the higher degree of differentiation occurs among regions of the non‐native range (average *F*ST _among regions_ = 0.258, average *F*ST _within regions_ = 0.008, t = 18.856 *p* < .001). Noteworthy, the two populations from China were significantly different from one another (*F*_ST_ = 0.188, *p* < .01). This differentiation at the native range carried into the invasive range. This was confirmed by the clustering dendrogram, where two highly differentiated branches, each harbouring a Chinese population and either one or two regions from the non‐native range, stem from a common ancestry (Figure S8).

Comparing pairwise differences between putative native sources and respective sinks in the invasive range revealed that locations within Turkey diverge more from Yangtze river than the Slovakian ones do from the Yellow river (average *F*ST _Turkey/Yangtze_ = 0.105, average *F*ST _Slovakia/Yellow_ = 0.05, t = 3.769 *p* = .032). Within Slovakia, the Vah river populations revealed to be less divergent from that of the Yellow river, although differentiation was not significant.

Inferences on possible hybridization revealed the existence of two pure lines whose individuals possessed a 100% pure genome. All individuals from Slovakia and the Yellow River (Pure 1) form the first line, while all individuals from Turkey and the Yangtze (Pure 2) belong to the second group. Interestingly, the large majority of individuals collected in France belong to Pure 1 genomic background and showed between 5%–75% of their genomes to have arisen from backcrosses. Noteworthy was a single individual exhibiting the genomic make‐up of a F1 (Figure S5).

### 3.4 Identification of candidate loci under selection at the invasive fronts

Data processing of Slovakian populations and subsequent match against common catalogue retrieved a total of 7,403 SNPs, the Turkish populations retrieved a total of 6,460 SNPs and the French populations retrieved a total of 4,556 SNPs. Inferences of candidate loci under selection via the two different procedures gave different quantitative and qualitative results. outflank detected a total of 19 candidate loci under selection in Slovakian populations, three in Turkish populations and none in the French populations. The allelic frequencies of a total of 30 loci correlated with river speed (for a BF > 10), one of which overlapped with outflank.

### 3.5 Candidate loci genetic diversity: Relationship among invasive regions in relation to native location

**Figure 5:**
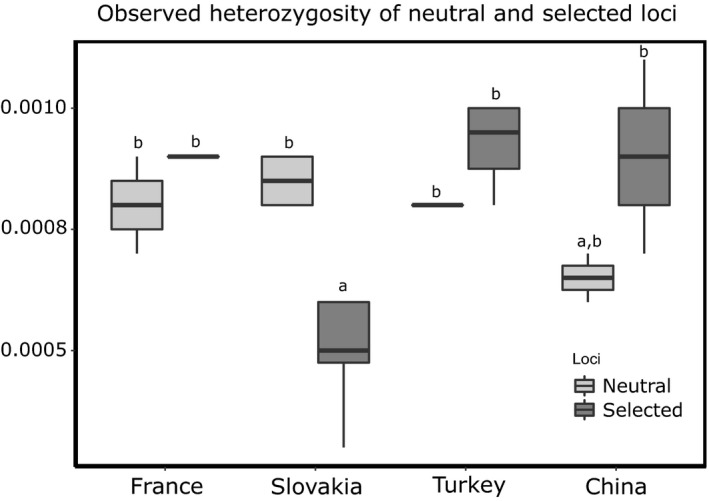
Comparison of observed heterozygosity of neutral and candidate loci among regions. Shifts on average observed heterozygosity, as signature of selective processes acting on standing genetic variation, are shown. On the *y*‐axis is represented observed heterozygosity (Ho), on the *x*‐axis, the respective region. Different colours in each boxplot represent whether it refers to neutral loci (labelled “neutrality”) or candidate loci (labelled “selection”). Shared letters on boxplots represent statistical nonsignificance after performing pairwise multiple comparisons with Tukey's HSD

The comparison of *Ho* between neutral and candidate loci across ranges and regions revealed a model including *region* fits better than a model including range as variable (ANOVA: *F_(model range vs model region)_* = 15.60; *df*_1,3_; *p* < .001, RSS model_region_ = 1E^−7^, model_range_ = 5E^−7^). Thus, a model with regions was preferred and proven to overall explain 71% of *Ho* variation (*R* ^2^ = .71; *F* = 12.18; *df*_7,24_; *p* < .001). Furthermore, it revealed a negative and significant effect of candidate loci in Slovakia (*t* = −5.45, *p* < .001) (Table S2), indicating that *Ho* is significantly reduced when compared to all others (Figure 5). Regarding private alleles, only locations in the native region were shown to possess them (CN‐YAN = 2, CN‐YEL = 3).

### 3.6 Candidate loci to discriminate population structure

We assumed that candidate loci could resolve fine‐scale population structure at least within the Slovakian region, where candidates were found. Clustering analyses revealed *K* = 2 as the model complexity maximizing marginal likelihood scores. AMOVA suggested that Slovakian, French and Turkish populations formed a single group while the Chinese populations of the Yellow river and of the Yangtze formed two independent groups (*F*_CT_ = 0.335, *p* < .001). DAPC analyses were less conclusive than those of neutral loci. Plotting the principal components for *K* = 3 (retaining 10 PCs and plotting two loadings (Figure S7b), as a term of comparison to neutral loci, revealed a more scattered graph where it is still possible to identify Turkish populations and respective source (China‐Yangtze River), while French, Slovakian and the Chinese population of the Yellow River grouped together (Figure 4b).

### 3.7 blast and gene ontology terms of candidate loci

Amongst the 21 candidates identified across invaded regions, seven detected in Slovakia showed a positive blast result against available fish genomes, supported by multiple species hits, amongst the most common were the cavefish (*Astyanax mexicanus*), codfish (*Gadus morhua*), zebrafish (*Danio rerio*) and the stickleback (*Gasterosteus aculeatus*) (Table 2). Biological processes were linked to negative regulation of cell cycle, protein transport and cell differentiation of eye and limb developmental processes. Blast against ncbi database produced two additional hits – one on the common carp *Cyprinus carpio* and one on a cyprinid endemic to the Chinese region of Yunan, *Sinocyclocheilus rhinocerous* ‐ associated with mRNA transcripts‐like involved in defence response to protozoan or regulation of T‐cell differentiation and glutathione processing (Table 3).

**Table 2:** Candidate loci alignment against teleost genomes

ensembl database							
Locus	Species		Length	Overlapping gene	Protein	GO terms	
						*Molecular function*	*Biological process*
1872	*Astyanax mexicanus*	Cave fish	32	*ppp1r13bb*	Protein phosphatase 1, regulatory subunit	Transcription binding factor	Apoptotic process/negative regulation of cell cycle
	*Danio rerio*	Zebrafish	65	*ppp1r13ba*			
			43	*ppp1r13bb*			
			37	*ppp1r13ba*			
2700	*Danio rerio*	Zebrafish	116	*v2rc1*	Vomeronasal 2 receptor, c1	G‐protein coupled receptor activity	G‐protein coupled receptor activity
3914	*Astyanax mexicanus*	Cave fish	83	*vps35*	Vacuolar protein sorting‐associated protein 35		Protein transport
	*Gasterosteus aculeatus*	Stickleback	89	*ENSGACG00000014193*			
	*Gadus morhua*	Codfish	83	*vps35*			
	*Latimeria chalumnae*	Coelacanth	78				
	*Lepisosteus oculatus*	Spotted gar	89	*vps35*			
	*Oreochromis niloticus*	Tilapia	91	*vps35*			
	*Poecilia formosa*	Amazon molly	77	*vps35*			
	*Takifugu rubripes*	Fugu	69	*ENSTRUG00000001105*			
4040	*Astyanax mexicanus*	Cave fish	109	*smoc1*	SPARC‐related modular calcium‐binding 1	Calcium ion binding extracellular matrix binding	Cell differentiation
	*Danio rerio*	Zebrafish	132	*smoc1*			
	*Gasterosteus aculeatus*	Stickleback	117	*smoc1*			
	*Gadus morhua*	Codfish	116				Limb development
	*Lepisosteus oculatus*	Spotted gar	97	*smoc1*			
	*Oryzias latipes*	Japanese medaka	28	*smoc*			
	*Oreochromis niloticus*	Tilapia	120	*smoc1*			
	*Poecilia formosa*	Amazon molly	28	*smoc1*			Regulation of osteoblast differentiation
	*Tetraodon nigroviridis*	Tetraodon	55	*smoc1*			
	*Takifugu rubripes*	Fugu	55	*smoc1*			
	*Xiphophorus maculatus*	Platyfish	52	*smoc1*			
4509	*Astyanax mexicanus*	Cave fish	51				
5822	*Astyanax mexicanus*	Cave fish	53	*ap5m1*	AP−5 complex subunit mu−1		Protein transport
	*Gasterosteus aculeatus*	Stickleback	65	*ap5m1*			
	*Gadus morhua*	Cod fish	48	*ap5m1*			
6535	*Danio rerio*	Zebrafish	100	*wnk1a*	WNK lysine deficient protein kinase 1a	ATP binding	Angiogenesis

**Table 3:** Candidate loci matches against ncbi database

ncbi database							
Locus	Species		Length	Overlapping gene	Protein	GO terms	
						Molecular function	Biological process
3405	*Sinocyclocheilus rhinocerous*		116	Interferon regulatory factor 4‐like	IRF4; Interferon regulatory factor 4	DNA‐binding transcription activator activity, RNA polymerase II‐specific	Defence response to protozoon
	*Cyprinus carpio*	Common Carp	116			Positive regulation of cold‐induced thermogenesis	Regulation of T‐helper cell differentiation
						Regulation of T‐helper cell differentiation	Cytokine‐mediated signalling pathway
3513	*Sinocyclocheilus rhinocerous*		115	5‐oxoprolinase‐like	OPLAH; 5‐oxoprolinase	Catalytic activity	Glutathione biosynthetic process
	*Ccarpio*	Common Carp	115				Glutathione metabolic process

## 4 DISCUSSION

### 4.1 Genomic diversity between native and invasive range

Population genetics of biological invasions has mostly been built around the paradigm of low diversity in non‐native populations as a consequence of a genetic bottleneck associated to founder effects (Allendorf & Lundquist, 2003). When comparing average observed heterozygosity estimates among invasive populations and those in the native range, we found that invasive populations have on average, a higher observed heterozygosity and a higher number of variant sites than those in the native range. Higher genetic diversity among invasive populations, however, is not an uncommon observation and is frequently attributed to multiple introductions from genetically distinct sources (Bock et al., 2015). Amongst the most well‐known cases are wetland grass (*Phalaris arundinacea*) introduced in North America from European native regions (Lavergne & Molofsky, 2007) or that of the brown anole lizard (*Anolis sagrei*), whose invasive populations in Florida were traced back to eight genetically distinct native sources (Kolbe et al., 2004). Even though higher genetic diversity among invasive *P. parva* populations has previously been reported, it was later attributed to an unbalanced sampling design lacking native populations (Hardouin et al., 2018; Simon et al., 2015). Here, our data mainly show a source‐sink population system. Because no admixture was detected, both the higher observed heterozygosity and number of variant sites observed are probably a result from a recovery of the expected genetic bottleneck associated with the introduction event. Regarding the number of private alleles, a higher number was detected in the native populations. This too reflects the relatively recent nature of the onset of introduction (~50 years). Native populations unique genetic diversity was lost in non‐native populations perhaps as the result of genetic drift commonly associated with founder effects (Estoup et al., 2016; Roman & Darling, 2007). A pattern of higher allelic or haplotypic diversity within the native range has been reported for instance in lionfish (*Pterois volitans* and *Pterois miles*) (Freshwater et al., 2009), blue spotted grouper (*Cephalopholis argus*) in Hawaii (Planes & Lecaillon, 1998) or cane toad (*Rhinella marina*) in Australia (Estoup, Wilson, Sullivan, Cornuet, & Moritz, 2001).

### 4.2 Signatures of the invasion process – disentangling introduction and dispersal

Visualization of clustering plots suggested the existence of three clusters, where France appears as a distinct group, while the other clusters correspond to Slovakian populations together with the Chinese population of the Yellow River and Turkish populations aggregated in the same cluster than the Chinese population of the Yangtze River. A likelihood of three clusters was further supported both by the DAPC analyses and by the AMOVA, though Slovakia and France alternatively split as the third cluster. The lack of full agreement among clustering analyses suggests an unresolved dispersal path in the invasive range for which post hoc analyses offered multiple solutions. On the one hand, degree of allelic frequency divergence of Slovakian and French populations against Yellow River points toward a genetically similar founding source. Here, individuals genetically similar to those from Slovakia/Yellow River could have been introduced in France, perhaps from Armenia, according to empirical record (Gozlan et al., 2010), as opposed to scenarios with a direct sourcing either from Slovakia or from the Yellow River. It is possible that the time since introduction, approximately 30 years, was not enough for divergence to conspicuously manifest at the genome‐wide level (Hey, 2006). On the other hand, hybridization inferences showed a high number of French individuals to be backcrosses of the pure line from which Slovakian and Yellow River specimens belong to. Two hypotheses might explain these patterns. The first considers the evolution in apparent isolation of French populations for some generations after being introduced in the system, after which new flux of individuals occurred probably from the original lineage present in Slovakia. Alternatively, because these fish are iteroparous and a putative small number of individuals was available for reproduction at the onset of the introduction, crosses could have occurred between parental and offspring generation (Streit, Städler, & Lively, 2013).

Regarding the Turkish populations, it is safe to consider that those were originally from a population genetically similar to that of the Yangtze catchment. The narrower allelic divergence and *F*_ST_ between Slovakian/Yellow River in comparison to Turkey/Yangtze River suggests that recent introductions have occurred homogenising genetic variance on the Slovakian/Yellow River axis. However, we cannot discard that Turkish populations here analysed are not as genetically closer to the Yangtze River as the Slovakians to the Yellow River.

### 4.3 Genomic diversity of candidate loci suggests selective pressures at a front

Screens for candidate loci under selection produced mostly results for the Slovakian region, perhaps due to the fact that sampled sites covered different freshwater systems. On the diversity of candidate loci, private alleles were only found in populations of the native range. This result not only confirms the pattern observed with neutral markers ‐ that native locations harbour original diversity but also reinforces the recent timing of introduction – no new alleles among the sampled invasive populations have been observed. It further indicates that selection might be acting on standing genetic variation, which is expected to be rapid in non‐native ranges (Prentis, Wilson, Dormontt, Richardson, & Lowe, 2008). Candidate loci in Slovakia exhibited a significantly lower heterozygosity than (a) neutral loci in the invasive range and (b) both neutral and candidate loci elsewhere, suggesting that selective sweeps have occurred in those genomic regions. Selective sweeps have also been identified among the populations of an invasive ascidian (Lin et al., 2017) and the Asian tiger mosquito (Goubert et al., 2017).

While range expansions are known to affect the genotypic composition of neutral standing genetic variation (Excoffier, Foll, & Petit, 2009), expectations for adaptive variation are extrapolated from heterozygosity‐fitness correlations (Peischl & Excoffier, 2015). If that is correct, adaptive loci are predicted to have a relatively weak effect on the individual fitness component (Peischl & Excoffier, 2015). Together with the recent time of the invasion, we argue that this could be the reason for the relatively low number of identified candidate loci.

Comparisons with whole genome databases of fish revealed a series of loci involved in key developmental processes, such as regulation of cell cycle, early stage development, protein transport or angiogenesis. In the context of biological invasions, candidate loci could be linked to the high plasticity in life history trait documented for topmouth gudgeon, where faster developmental rates upon the introduction in a novel habitat would facilitate establishment by rapidly increased density. Of similar significance are loci related to mRNA‐like transcripts of two cyprinids. One is that of glutathione an antioxidant involved in organismal response to heavy metal exposure (Regoli & Principato, 1995; Ren et al., 2002; Timofeyev et al., 2004). Danube tributaries in the region of former Czechoslovakia, and specifically the river Vah, are documented to be polluted with heavy metal concentrations and chemicals released from the paper industry (Füllenbach, 2017; Gondová, Janiga, Hundža, & Solár, 2017). It is possible that in this case, pollution indicators could be a factor covarying with river flow speed where for instances more stagnant waters – which this species favours – tend to accumulate more pollutants residues over time. The other locus matched a mRNA transcript associated with adaptive immune response. Given the relationship between topmouth gudgeon and the rosette agent *Sphaerothecum destruens* for which the topmouth gudgeon is a healthy carrier (Andreou & Gozlan, 2016), the identification of loci associated with immune response could be a signature of the fish immunocompetence, further facilitating the species’ establishment among native fish communities (Combe & Gozlan, 2018).

### 4.4 Candidate loci to discriminate population structure

No evidence for fine‐scale population structure was found with candidate loci. The role of putative candidates in defining fine‐scale population structure is probably hampered by the relatively young age of invasive populations. While recent works suggest that candidate loci increase the resolution at which population structure can be detected, i.e., hake (*Merluccius merluccius*) in the Mediterranean (Milano et al., 2014) or the Eastern Atlantic wrasse (*Symphodus tinca*) (Carreras et al., 2017), it may, however, not apply to newly established systems. Noteworthy, and contrary to what was observed with the full data set, molecular variance among candidate loci discriminated the native from the invasive range, suggesting that divergence at these loci is accumulating at a faster pace than that of the background genome. The observation that candidate loci can discriminate between native and non‐native range suggests that this type of markers are potentially effective to document biological invasions whose history is not as well documented as that of the topmouth gudgeon.

In conclusion, the detection of occurrence and spread of biological invaders are dependent on human observation and documentation. Population genetics provide temporal and spatial depth to those observations and enable the characterization of invasion pathways, dispersal and establishment in the new range (Estoup & Guillemaud, 2010). Our study describes the genomic signature in one of the most successful fish invaders. We show that genetically dissimilar source populations, each with specific evolutionary histories, could prompt distinct genomic response of adaptation. Disentangling whether related to selective pressures in the introduced environments or to methodological caveats linked to recent divergence times, is open to future exploration. Nevertheless, considering the role of adaptive genetic variation in establishment success, we uphold the suggestion that factors underlying successful invasions should be decomposed at the population level. Together, we show that evolutionary histories in native and non‐native ranges determine the genetic make‐up of invasive populations contributing to their adaptive potential and successful establishment.

## AUTHOR CONTRIBUTIONS

M.B.S. performed research, analysed the data and wrote the manuscript; S.B., J.C., and R.E.G. designed research and contributed with data; A.S.T., E.Z. contributed with data; C.E. designed research, assisted with analyses and writing the manuscript. S.B., J.C., and C.E. received funding for this research from BIODIVERSA.

## Data Availability

Raw data is available under the project number PRJNA560205 at NCBI's SRA. VCF files with genomic data utilized in this work are uploaded on DRYAD: https://doi.org/10.5061/dryad.98sf7m0f0
