# Supplementary figures and images for: Genomic footprints of a biological invasion: Introduction from Asia and dispersal in Europe of the topmouth gudgeon (Pseudorasbora parva)
Source: Mol Ecol. 2019 Dec 10;29(1):71–85. doi: 10.1111/mec.15313 (PMC7003831; doi:10.1111/mec.15313)

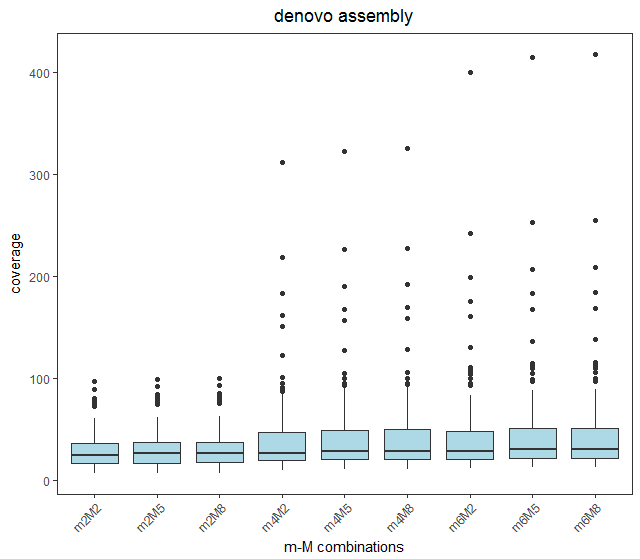

Supplement: Supplementary file 1 [file MEC-29-71-s001.png]

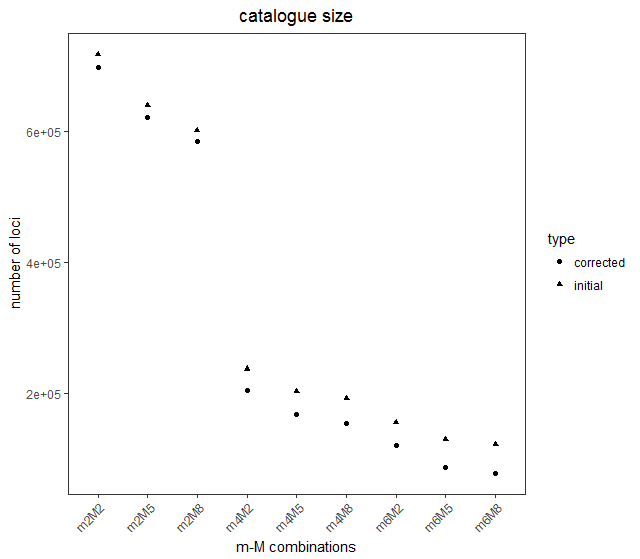

Supplement: Supplementary file 2 [file MEC-29-71-s002.png]

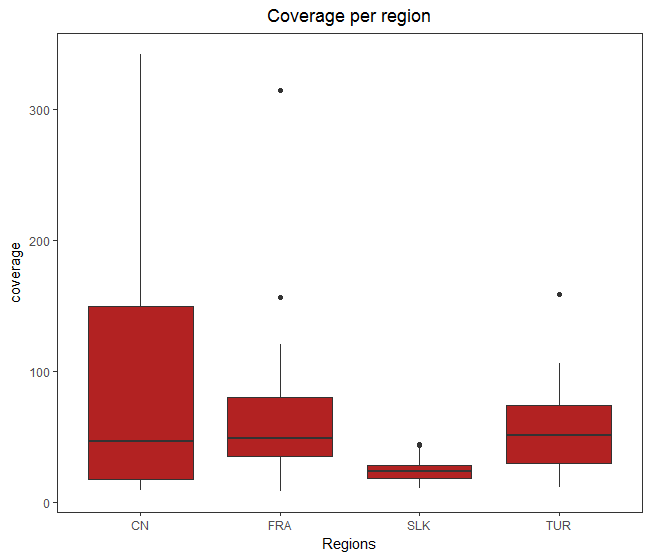

Supplement: Supplementary file 3 [file MEC-29-71-s003.png]

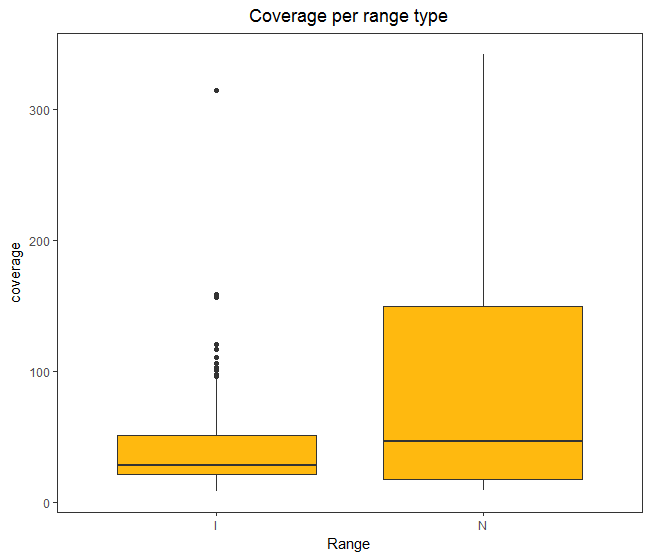

Supplement: Supplementary file 4 [file MEC-29-71-s004.png]

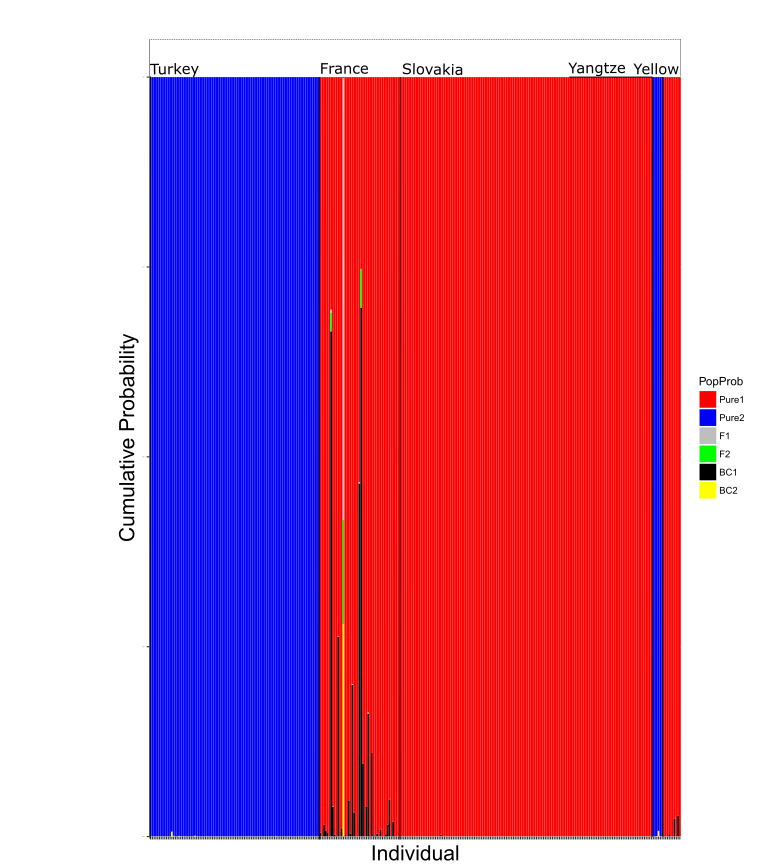

Supplement: Supplementary file 5 [file MEC-29-71-s005.png]

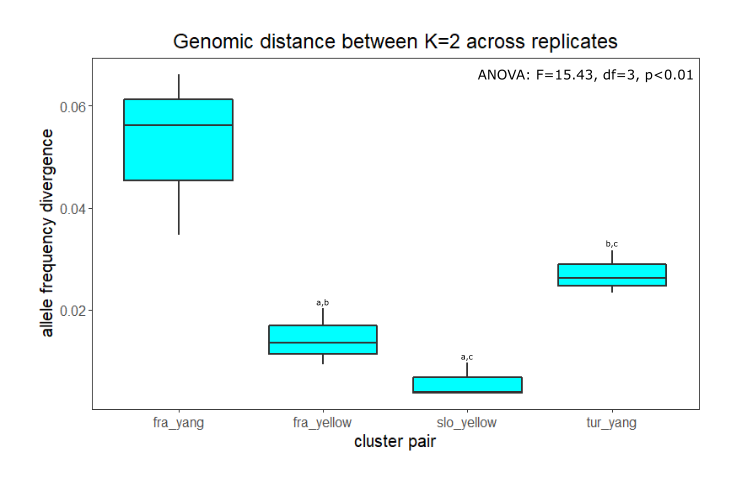

Supplement: Supplementary file 6 [file MEC-29-71-s006.png]

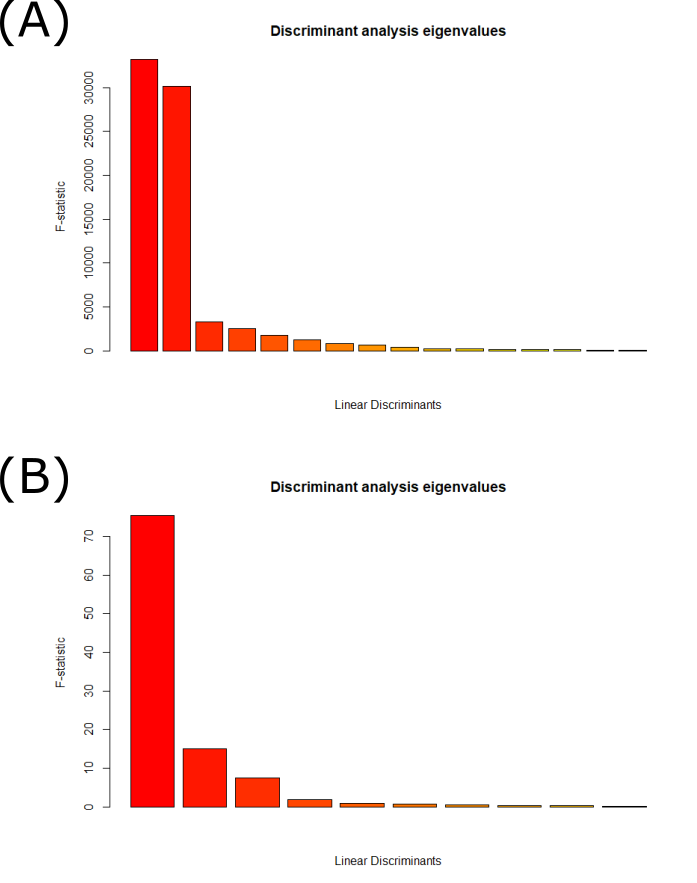

Supplement: Supplementary file 7 [file MEC-29-71-s007.png]

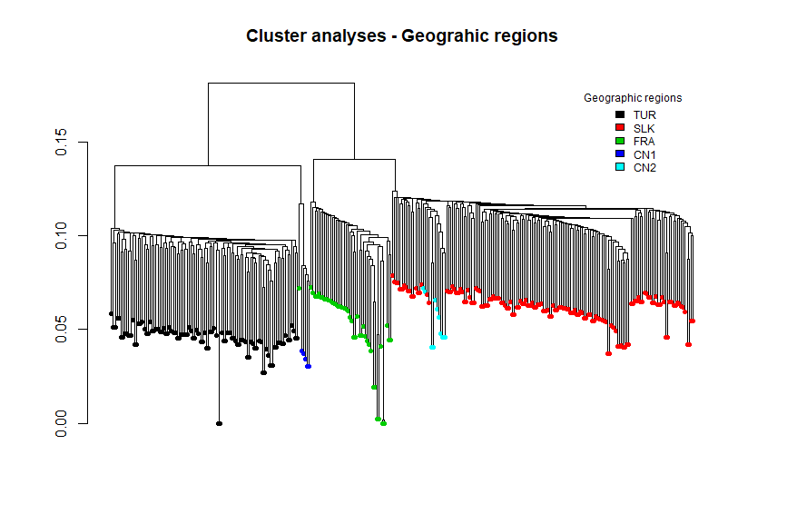

Supplement: Supplementary file 8 [file MEC-29-71-s008.png]
